# The predictive power of data: machine learning analysis for Covid-19 mortality based on personal, clinical, preclinical, and laboratory variables in a case–control study

**DOI:** 10.1186/s12879-024-09298-w

**Published:** 2024-04-18

**Authors:** Maryam Seyedtabib, Roya Najafi-Vosough, Naser Kamyari

**Affiliations:** 1 Department of Biostatistics and Epidemiology, School of Health, Ahvaz Jundishapur University of Medical Sciences, Ahvaz, Iran; 2 Research Center for Health Sciences, Hamadan University of Medical Sciences, Hamadan, Iran; 3 Department of Biostatistics and Epidemiology, School of Health, Abadan University of Medical Sciences, Abadan, Iran

**Keywords:** Predictive model, Coronavirus disease, Machine learning, Data quality, Performance

## Abstract

**Background and purpose:**

The COVID-19 pandemic has presented unprecedented public health challenges worldwide. Understanding the factors contributing to COVID-19 mortality is critical for effective management and intervention strategies. This study aims to unlock the predictive power of data collected from personal, clinical, preclinical, and laboratory variables through machine learning (ML) analyses.

**Methods:**

A retrospective study was conducted in 2022 in a large hospital in Abadan, Iran. Data were collected and categorized into demographic, clinical, comorbid, treatment, initial vital signs, symptoms, and laboratory test groups. The collected data were subjected to ML analysis to identify predictive factors associated with COVID-19 mortality. Five algorithms were used to analyze the data set and derive the latent predictive power of the variables by the shapely additive explanation values.

**Results:**

Results highlight key factors associated with COVID-19 mortality, including age, comorbidities (hypertension, diabetes), specific treatments (antibiotics, remdesivir, favipiravir, vitamin zinc), and clinical indicators (heart rate, respiratory rate, temperature). Notably, specific symptoms (productive cough, dyspnea, delirium) and laboratory values (D-dimer, ESR) also play a critical role in predicting outcomes. This study highlights the importance of feature selection and the impact of data quantity and quality on model performance.

**Conclusion:**

This study highlights the potential of ML analysis to improve the accuracy of COVID-19 mortality prediction and emphasizes the need for a comprehensive approach that considers multiple feature categories. It highlights the critical role of data quality and quantity in improving model performance and contributes to our understanding of the multifaceted factors that influence COVID-19 outcomes.

**Supplementary Information:**

The online version contains supplementary material available at 10.1186/s12879-024-09298-w.

## Introduction

The World Health Organization (WHO) has declared COVID-19 a global pandemic in March 2020 [1]. The first cases of SARSCoV-2, a new severe acute respiratory syndrome coronavirus, were detected in Wuhan, China, and rapidly spread to become a global public health problem [2]. The clinical presentation and symptoms of COVID-19 may be similar to those of Middle East Respiratory Syndrome (MERS) and Severe Acute Respiratory Syndrome (SARS), however the rate of spread is higher [3]. By December 31, 2022, the pandemic had caused more than 729 million cases and nearly 6.7 million deaths (0.92%) were confirmed in 219 countries worldwide [4]. For many countries, figuring out what measures to take to prevent death or serious illness is a major challenge. Due to the complexity of transmission and the lack of proven treatments, COVID-19 is a major challenge worldwide [5, 6]. In middle- and low-income countries, the situation is even more catastrophic due to high illiteracy rates, a very poor health care system, and lack of intensive care units [5]. In addition, understanding the factors contributing to COVID-19 mortality is critical for effective management and intervention strategies [6].

Numerous studies have shown several factors associated with COVID-19 outcomes, including socioeconomic, environmental, individual demographic, and health factors [7–9]. Risk factors for COVID -19 mortality vary by study and population studied [10]. Age [11, 12], comorbidities such as hypertension, cardiovascular disease, diabetes, and COPD [13–15], sex [13], race/ethnicity [11], dementia, and neurologic disease [16, 17], are some of the factors associated with COVID-19 mortality. Laboratory factors such as elevated levels of inflammatory markers, lymphopenia, elevated creatinine levels, and ALT are also associated with COVID-19 mortality [5, 18]. Understanding these multiple risk factors is critical to accurately diagnose and treat COVID-19 patients.

Accurate diagnosis and treatment of the disease requires a comprehensive assessment that considers a variety of factors. These factors include personal factors such as medical history, lifestyle, and genetics; clinical factors such as observations on physical examinations and physician reports; preclinical factors such as early detection through screening or surveillance; laboratory factors such as results of diagnostic tests and medical imaging; and patient-reported signs and symptoms. However, the variety of characteristics associated with COVID-19 makes it difficult for physicians to accurately classify COVID-19 patients during the pandemic.

In today's digital transformation era, machine learning plays a vital role in various industries, including healthcare, where substantial data is generated daily [19–21]. Numerous studies have explored machine learning (ML) and explainable artificial intelligence (AI) in predicting COVID-19 prognosis and diagnosis [22–25]. Chadaga et al. have developed decision support systems and triage prediction systems using clinical markers and biomarkers [22, 23]. Similarly, Khanna et al. have developed a ML and explainable AI system for COVID-19 triage prediction [24]. Zoabi has also made contributions in this field, developing ML models that predict COVID-19 test results with high accuracy based on a small number of features such as gender, age, contact with an infected person and initial clinical symptoms [25]. These studies emphasize the potential of ML and explainable AI to improve COVID-19 prediction and diagnosis. Nonetheless, the efficacy of ML algorithms heavily relies on the quality and quantity of data utilized for training. Recent research has indicated that deep learning algorithms' performance can be significantly enhanced compared to traditional ML methods by increasing the volume of data used [26]. However, it is crucial to acknowledge that the impact of data volume on model performance can vary based on data characteristics and experimental setup, highlighting the need for careful consideration and analysis when selecting data for model training. While the studies emphasize the importance of features in training ML algorithms for COVID-19 prediction and diagnosis, additional research is required on methods to enhance the interpretability of features.

Therefore, the primary aim of this study is to identify the key factors associated with mortality in COVID -19 patients admitted to hospitals in Abadan, Iran. For this purpose, seven categories of factors were selected, including demographic, clinical and conditions, comorbidities, treatments, initial vital signs, symptoms, and laboratory tests, and machine learning algorithms were employed. The predictive power of the data was assessed using 139 predictor variables across seven feature sets. Our next goal is to improve the interpretability of the extracted important features. To achieve this goal, we will utilize the innovative SHAP analysis, which illustrates the impact of features through a diagram.

## Materials and methods

### Study population and data collection

Using data from the COVID-19 hospital-based registry database, a retrospective study was conducted from April 2020 to December 2022 at Ayatollah Talleghani Hospital (a COVID‑19 referral center) in Abadan City, Iran.

**Fig. 1:**
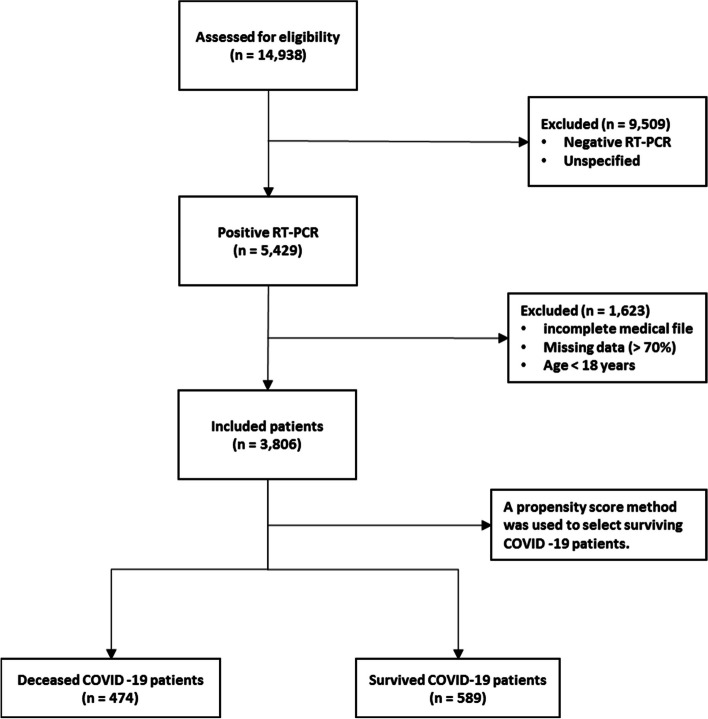
Flowchart describing the process of patient selection

A total of 14,938 patients were initially screened for eligibility for the study. Of these, 9509 patients were excluded because their transcriptase polymerase chain reaction (RT-PCR) test results were negative or unspecified. The exclusion of patients due to incomplete or missing data is a common issue in medical research, particularly in the use of electronic medical records (EMRs) [27]. In addition, 1623 patients were excluded because their medical records contained more than 70% incomplete or missing data. In addition, patients younger than 18 years were not included in the study. The criterion for excluding 1623 patients due to "70% incomplete or missing data" means that the medical records of these patients did not contain at least 30% of the data required for a meaningful analysis. This threshold was set to ensure that the dataset used for the study contained a sufficient amount of complete and reliable information to draw accurate conclusions. Incomplete or missing data in a medical record may relate to key variables such as patient demographics, symptoms, lab results, treatment information, outcomes, or other data points important to the research. Insufficient data can affect the validity and reliability of study results and lead to potential bias or inaccuracies in the findings. It is important to exclude such incomplete records to maintain the quality and integrity of the research findings and to ensure that the conclusions drawn are based on robust and reliable data. After these exclusions, 3806 patients remained. Of these patients, 474 died due to COVID -19, while the remaining 3332 patients recovered and were included in the control group. To obtain a balanced sample, the control group was selected with a propensity score matching (PSM). The PSM refers to a statistical technique used to create a balanced comparison group by matching individuals in the control group (in this case, the survived group) with individuals in the case group (in this case, the deceased group) based on their propensity scores. In this study, the propensity scores for each person represented the probability of death (coded as a binary outcome; survived = 0, deceased = 1) calculated from a set of covariates (demographic factors) using the matchit function from the MatchIt library. Two individuals, one from the deceased group and one from the survived group, are considered matched if the difference between their propensity scores is small. Non-matching participants are discarded. The matching aims to reduce bias by making the distribution of observed characteristics similar between groups, which ultimately improves the comparability of groups in observational studies [28]. In total, the study included 1063 COVID-19 patients who belonged to either the deceased group (case = 474) or the survived group (control = 589) (Fig. 1).

In the COVID‑19 hospital‑based registry database, one hundred forty primary features in eight main classes including patient’s demographics (eight features), clinical and conditions features (16 features), comorbidities (18 features), treatment (17 features), initial vital sign (14 features), symptoms during hospitalization (31 features), laboratory results (35 features), and an output (0 for survived and 1 for deceased) was recorded for COVID-19 patients. The main features included in the hospital-based COVID-19 registry database are provided in Appendix Table 1.

**Table 1:** Baseline characteristics of patients infected with COVID-19

Demographics data		Total (*n* = 1063)	Survived (*n* = 589)	Deceased (*n* = 474)	*P*-value
Age, year		59.53 ± 16.32	54.70 ± 15.60	65.53 ± 15.18	< 0.001^†^
	< 40 Y	161 (15.1%)	124 (77.0%)	37 (23.0%)	< 0.001^‡^
	40 – 60 Y	379 (35.7%)	254 (67.0%)	125 (33.0%)	
	> 60 Y	523 (49.2%)	211 (40.3%)	312 (59.7%)	
Sex	Male	822 (77.3%)	455 (55.4%)	367 (44.6%)	0.946^‡^
	Female	241 (22.7%)	134 (55.6%)	107 (44.4%)	
Occupation	Unemployed	290 (26.5%)	160 (55.2%)	130 (44.8%)	0.265^‡^
	Employee	102 (9.7%)	49 (48.0%)	53 (52.0%)	
	Self-Employment	671 (38.5%)	380 (56.6%)	291 (43.4%)	
Place of residence	Urban	796 (74.9%)	437 (54.9%)	359 (45.1%)	0.564^‡^
	Rural	267 (25.1%)	152 (56.9%)	115 (43.1%)	
Marital status	Married	813 (76.5%)	460 (56.6%)	353 (43.4%)	0.166^‡^
	Single	250 (23.5%)	129 (51.6%)	121 (48.4%)	
Education level	Under diploma	793 (74.6%)	422 (53.2%)	371 (46.8%)	0.017^‡^
	Undergraduate	258 (24.3%)	162 (62.8%)	96 (37.2%)	
	Postgraduate	12 (1.1%)	5 (41.7%)	7 (58.3%)	
BMI (kg/cm^2^)	< 18	27 (2.5%)	7 (25.9%)	20 (74.1%)	< 0.001^‡^
	18 – 25	603 (56.7%)	370 (61.4%)	233 (38.6%)	
	26 – 30	210 (19.8%)	115 (54.8%)	95 (45.2%)	
	> 30	223 (21.0%)	97 (43.5%)	126 (56.5%)	
Season of admission	Spring	272 (25.6%)	154 (56.6%)	118 (43.4%)	0.785^‡^
	Summer	238 (22.4%)	134 (56.3%)	104 (43.7%)	
	Autumn	315 (29.6%)	176 (55.9%)	139 (47.5%)	
	Winter	238 (22.4%)	125 (52.5%)	113 (23.5%)	

^†^ *P*-value conducted from Independent t-test

^‡^ *P*-value conducted from Chi-square test

To ensure the accuracy of the recorded information, discharged patients or their relatives were called and asked to review some of the recorded information (demographic information, symptoms, and medical history). Clinical symptoms and vital signs were referenced to the first day of hospitalization (at admission). Laboratory test results were also referenced to the patient’s first blood sample at the time of hospitalization.

The study analyzed 140 variables in patients' records, normalizing continuous variables and creating a binary feature to categorize patients based on outcomes. To address the issue of an imbalanced dataset, the Synthetic Minority Over-sampling Technique (SMOTE) was utilized. Some classes were combined to simplify variables. For missing data, an imputation technique was applied, assuming a random distribution [29]. Little's MCAR test was performed with the naniar package to assess whether missing data in a dataset is missing completely at random (MCAR) [30]. The null hypothesis in this test is that the data are MCAR, and the test statistic is a chi-square value.

The Ethics Committee of Abadan University of Medical Science approved the research protocol (No. IR.ABADANUMS.REC.1401.095).

### Predictor variables

All data were collected in eight categories, including demographic, clinical and conditions, comorbidities, treatment, initial vital signs, symptoms, and laboratory tests in medical records, for a total of 140 variables.

The "Demographics" category encompasses eight features, three of which are binary variables and five of which are categorical. The "Clinical Conditions" category includes 16 features, comprising one quantitative variable, 12 binary variables, and five categorical features. "Comorbidities", "Treatment", and "Symptoms" each have 18, 17, and 30 binary features, respectively. Also, there is one quantitative variable in symptoms category. The "Initial Vital Signs" category features 11 quantitative variables, two binary variables, and one categorical variable. Finally, the "Laboratory Tests" category comprises 35 features, with 33 being quantitative, one categorical, and one binary (Appendix Table 1).

### Outcome variable

The primary outcome variable was mortality, with December 31, 2022, as the last date of follow‐up. The feature shows the class variable, which is binary. For any patient in the survivor group, the outcome is 0; otherwise, it is 1. In this study, 44.59% (*n* = 474) of the samples were in the deceased group and were labeled 1.

### Data balancing

In case–control studies, it is common to have unequal size groups since cases are typically fewer than controls [31]. However, in case–control studies with equal sizes, data balancing may not be necessary for ML algorithms [32]. When using ML algorithms, data balancing is generally important when there is an imbalance between classes, i.e., when one class has significantly fewer observations than the other [33]. In such cases, balancing can improve the performance of the algorithm by reducing the bias in favor of the majority class [34]. For case–control studies of the same size, the balance of the classes has already been reached and balancing may not be necessary. However, it is always recommended to evaluate the performance of the ML algorithm with the given data set to determine the need for data balancing. This is because unbalanced case–control ratios can cause inflated type I error rates and deflated type I error rates in balanced studies [35].

### Feature selection

Feature selection is about selecting important variables from a large dataset to be used in a ML model to achieve better performance and efficiency. Another goal of feature selection is to reduce computational effort by eliminating irrelevant or redundant features [36, 37]. Before generating predictions, it is important to perform feature selection to improve the accuracy of clinical decisions and reduce errors [37]. To identify the best predictors, researchers often compare the effectiveness of different feature selection methods. In this study, we used five common methods, including Decision Tree (DT), eXtreme Gradient Boosting (XGBoost), Support Vector Machine (SVM), Naïve Bayes (NB), and Random Forest (RF), to select relevant features for predicting mortality of COVID -19 patients. To avoid overfitting, we performed ten-fold cross-validation when training our dataset. This approach may help ensure that our model is optimized for accurate predictions of health status in COVID -19 patients.

### Model development, evaluation, and clarity

In this study, the predictive models were developed with five ML algorithms, including DT, XGBoost, SVM, NB, and RF, using the R programming language (v4.3.1) and its packages [38]. We used cross-validation (CV) to tune the hyperparameters of our models based on the training subset of the dataset. For training and evaluating our ML models, we used a common technique called tenfold cross validation [39]. The primary training dataset was divided into ten folding, each containing 10% of the total data, using a technique called stratified random sampling. For each of the 30% of the data, a ML model was built and trained on the remaining 70% of the data. The performance of the model was then evaluated on the 30%-fold sample. This process was repeated 100 times with different training and test combinations, and the average performance was reported.

Performance measures include sensitivity (recall), specificity, accuracy, F1-score, and the area under the receiver operating characteristics curve (AUC ROC). Sensitivity is defined as TP / (TP + FN), whereas specificity is TN / (TN + FP). F1-score is defined as the harmonic mean of Precision and Recall with equal weight, where Precision equals TP + TN / total. Also, AUC refers to the area under the ROC curve. In the evaluation of ML techniques, values were classified as poor if below 50%, ok if between 50 and 80%, good if between 80 and 90%, and very good if greater than 90%. These criteria are commonly used in reporting model evaluations [40, 41].

Finally, the shapely additive explanation (SHAP) method was used to provide clarity and understanding of the models. SHAP uses cooperative game theory to determine how each feature contributes to the prediction of ML models. This approach allows the computation of the contribution of each feature to model performance [42, 43]. For this purpose, the package shapr was used, which includes a modified iteration of the kernel SHAP approach that takes into account the interdependence of the features when computing the Shapley values [44].

## Results

### Patient characteristics

Table 1 shows the baseline characteristics of patients infected with COVID-19, including demographic data such as age and sex and other factors such as occupation, place of residence, marital status, education level, BMI, and season of admission. A total of 1063 adult patients (≥ 18 years) were enrolled in the study, of whom 589 (55.41%) survived and 474 (44.59%) died. Analysis showed that age was significantly different between the two groups, with a mean age of 54.70 ± 15.60 in the survivor group versus 65.53 ± 15.18 in the deceased group (*P* < 0.001). There was also a significant association between age and survival, with a higher proportion of patients aged < 40 years in the survivor group (77.0%) than in the deceased group (23.0%) (*P* < 0.001). No significant differences were found between the two groups in terms of sex, occupation, place of residence, marital status, and time of admission. However, there was a significant association between educational level and survival, with a lower proportion of patients with a college degree in the deceased group (37.2%) than in the survivor group (62.8%) (*P* = 0.017). BMI also differed significantly between the two groups, with the proportion of patients with a BMI > 30 (kg/cm^2^) being higher in the deceased group (56.5%) than in the survivor group (43.5%) (*P* < 0.001).

### Clinical and conditions

Important insights into the various clinical and condition characteristics associated with COVID-19 infection outcomes provides in Table 2. The results show that patients who survived the infection had a significantly shorter hospitalization time (2.20 ± 1.63 days) compared to those who died (4.05 ± 3.10 days) (*P* < 0.001). Patients who were admitted as elective cases had a higher survival rate (84.6%) compared to those who were admitted as urgent (61.3%) or emergency (47.4%) cases. There were no significant differences with regard to the number of infections or family infection history. However, patients who had a history of travel had a lower decease rate (40.1%).

**Table 2:** Clinical and conditions characteristics of patients infected with COVID-19

Clinical and conditions data		Total (*n* = 1063)	Survived (*n* = 589)	Deceased (*n* = 474)	*P*-value
Hospitalization, day		3.02 ± 2.57	2.20 ± 1.63	4.05 ± 3.10	< 0.001^†^
Admission type	Emergency	561 (52.8%)	266 (47.4%)	295 (52.6%)	< 0.001^‡^
	Urgent	437 (41.1%)	268 (61.3%)	169 (38.7%)	
	Elective	65 (6.1%)	55 (84.6%)	10 (15.4%)	
No. of infection	Once	756 (71.1%)	423 (56.0%)	333 (44.0%)	0.673^‡^
	Twice	249 (23.4%)	137 (55.0%)	112 (45.0%)	
	Three or more	58 (5.5%)	29 (50.0%)	29 (50.0%)	
Family infection	Yes	798 (75.1%)	442 (55.4%)	356 (44.6%)	0.981^‡^
Travel	Yes	451 (42.4%)	270 (59.9%)	181 (40.1%)	0.012^‡^
Communication	Yes	787 (74.0%)	433 (55.0%)	354 (45.0%)	0.666^‡^
CPR case	Yes	358 (33.7%)	162 (45.3%)	196 (54.7%)	< 0.001^‡^
Underlying conditions	Yes	389 (36.6%)	149 (38.3%)	240 (61.7%)	< 0.001^‡^
Hyperlipidemia	Yes	199 (18.7%)	46 (23.1%)	153 (76.9%)	< 0.001^‡^
Alcohol consumption	Yes	16 (1.5%)	2 (12.5%)	14 (87.5%)	< 0.001^‡^
Transplantation	Yes	20 (1.9%)	6 (30.0%)	14 (70.0%)	0.021^‡^
Chemotropic	Yes	14 (1.3%)	3 (21.4%)	11 (78.6%)	0.010^‡^
Special Drugs	Yes	8 (0.8%)	0 (0.0%)	8 (100%)	0.002^‡^
Immunosuppressive Drugs	Yes	40 (3.8%)	12 (30.0%)	28 (70.0%)	< 0.001^‡^
Pregnancy	Yes	9 (0.8%)	4 (44.4%)	5 (55.6%)	0.506^‡^
Smoking	Recently	365 (34.3%)	133 (36.4%)	232 (63.6%)	< 0.001^‡^
	Before	174 (16.4%)	97 (55.7%)	77 (44.3%)	
	Never	524 (49.3%)	359 (68.5%)	165 (31.5%)	

*CPR* Cardiopulmonary Resuscitation

^**†**^ *P*-value conducted from Independent t-test

^**‡**^ *P*-value conducted from Chi-square test

A significantly higher proportion of deceased patients had cases requiring CPR (54.7% vs. 45.3%). Patients who had underlying medical conditions had a significantly lower survival rate (38.3%), with hyperlipidemia being the most prevalent condition (18.7%). Patients who had a history of alcohol consumption (12.5%), transplantation (30.0%), chemotropic (21.4%) or special drug use (0.0%), and immunosuppressive drug use (30.0%) also had a lower survival rate. Pregnant patients (44.4%) had similar survival outcomes compared to non-pregnant patients (55.6%). Patients who were recent or current smokers (36.4%) also had a significantly lower survival rate.

### Comorbidities

Table 3 summarizes the comorbidity characteristics of COVID-19 infected patients. Out of 1063 patients, 54.84% had comorbidities. Chi-Square tests for individual comorbidities showed that most of them had a significant association with COVID-19 outcomes, with *P*-values less than 0.05. Among the various comorbidities, hypertension (HTN) and diabetes mellitus (DM) were the most prevalent, with 12% and 11.5% of patients having these conditions, respectively. The highest fatality rates were observed among patients with cardiovascular disease (95.5%), chronic kidney disease (62.5%), gastrointestinal (GI) (93.3%), and liver diseases (73.3%). Conversely, patients with neurology comorbidities had the lowest fatality rate (0%). These results highlight the significant role of comorbidities in COVID-19 outcomes and emphasize the need for special attention to be paid to patients with pre-existing health conditions.

**Table 3:** Comorbidities characteristics of patients infected with COVID-19

Comorbidities data		Total (*n* = 1063)	Survived (*n* = 589)	Deceased (*n* = 474)	*P*-value
Comorbidity	Yes	583 (54.84)	182 (31.3%)	401 (68.7%)	< 0.001^‡^
HTN	Yes	128 (12.0%)	28 (21.9%)	100 (78.1%)	< 0.001^‡^
DM	Yes	122 (11.5%)	22 (18.0%)	100 (82.0%)	< 0.001^‡^
CVD	Yes	44 (4.1%)	2 (4.5%)	42 (95.5%)	< 0.001^‡^
CKD	Yes	40 (3.8%)	15 (37.5%)	25 (62.5%)	0.020^‡^
COPD	Yes	15 (1.4%)	0 (0.0%)	15 (100%)	0.115^‡^
HIV	Yes	2 (0.2%)	0 (0.0%)	2 (100%)	< 0.001^‡^
HBV	Yes	5 (0.5%)	0 (0.0%)	5 (100%)	0.012^‡^
Cancer	Yes	17 (1.6%)	3 (17.6%)	14 (82.4%)	0.002^‡^
Respiratory	Yes	30 (2.8%)	2 (6.7%)	28 (93.3%)	< 0.001^‡^
GI	Yes	49 (4.6%)	46 (93.9%)	3 (6.1%)	< 0.001^‡^
Neurology	Yes	7 (0.7%)	7 (100%)	0 (0.0%)	0.017^‡^
Endocrine	Yes	18 (1.7%)	8 (44.4%)	10 (55.6%)	0.345^‡^
Liver	Yes	30 (2.8%)	8 (26.7%)	22 (73.3%)	0.001^‡^
Hematology	Yes	4 (0.4%)	0 (0.0%)	4 (100%)	0.026^‡^
Dermatology	Yes	34 (3.2%)	12 (35.3%)	22 (64.7%)	0.016^‡^
Psychology	Yes	5 (0.5%)	1 (20.0%)	4 (80.0%)	0.110^‡^
Other diseases	Yes	33 (3.1%)	19 (57.6%)	14 (42.4%)	0.799^‡^

*HTN* Hypertension, *DM* Diabetes mellitus, *CVD* Cardiovascular disease, *CKD* Chronic kidney disease, *COPD* Chronic obstructive pulmonary disease, *HIV* Human immunodeficiency virus, *HBV* Hepatitis B virus, *Respiratory* Such as influenza, pneumonia, asthma, bronchitis, and chronic obstructive airways disease, *GI* Gastrointestinal, *Neurology* Such as epilepsy, learning disabilities, neuromuscular disorders, autism, ADD, brain tumors, and cerebral palsy, *Liver* Such as fatty liver disease and cirrhosis, *Hematology* Blood disease, *Dermatology* Skin diseases, *Psychology* Mental disorders

^‡^: *P*-value conducted from Chi-square test

### Treatment

The treatment characteristics of the COVID-19 patients and the resulting outcomes are shown in Table 4. The table shows the frequency of patients who received different types of medications or therapies during their treatment. According to the results, the use of antibiotics (35.1%), remdesivir (29.6%), favipiravir (36.0%), and Vitamin zinc (33.5%) was significantly associated with a lower mortality rate (*P* < 0.001), suggesting that these medications may have a positive impact on patient outcomes. On the other hand, the use of Heparin (66.1%), Insulin (82.6%), Antifungal (89.6%), ACE inhibitors (78.1%), and Angiotensin II Receptor Blockers (ARB) (83.8%) was significantly associated with increased mortality (*P* < 0.001), suggesting that these medications may have a negative effect on the patient's outcome. Also, It seems that taking hydroxychloroquine (51.0%) is associated with a worse outcome at lower significance (*P* = 0.022). The use of Atrovent, Corticosteroids and Non-Steroidal Anti-Inflammatory Drugs (NSAIDs) did not show a significant association with survival or mortality rates. Similarly, the use of Intravenous Immunoglobulin (IVIg), Vitamin C, Vitamin D, and Diuretic did not show a significant association with the patient’s outcome.

**Table 4:** Treatment characteristics of patients infected with COVID-19

Treatment data		Total (*n* = 1063)	Survived (*n* = 589)	Deceased (*n* = 474)	*P*-value
Antibiotic	Yes	439 (41.3%)	285 (64.9%)	154 (35.1%)	< 0.001^‡^
Remdesivir	Yes	476 (44.8%)	335 (70.4%)	141 (29.6%)	< 0.001^‡^
Favipiravir	Yes	572 (53.8%)	366 (64.0%)	206 (36.0%)	< 0.001^‡^
Hydroxychloroquine	Yes	241 (22.7%)	118 (49.0%)	123 (51.0%)	0.022^‡^
Heparin	Yes	171 (16.1%)	58 (33.9%)	113 (66.1%)	< 0.001^‡^
Atrovent	Yes	50 (4.7%)	21 (42.0%)	29 (58.0%)	0.051^‡^
Insulin	Yes	109 (10.3%)	19 (17.4%)	90 (82.6%)	< 0.001^‡^
Diuretic	Yes	95 (8.9%)	57 (60.0%)	38 (40.0%)	0.346^‡^
Antifungal	Yes	251 (23.6%)	26 (10.4%)	225 (89.6%)	< 0.001^‡^
Corticosteroid	Yes	933 (87.8%)	517 (55.4%)	416 (44.6%)	0.995^‡^
IVIg	Yes	77 (7.2%)	39 (50.6%)	38 (49.4%)	0.383^‡^
NSAIDs	Yes	815 (76.7%)	450 (55.2%)	365 (44.8%)	0.817^‡^
ACEi	Yes	128 (12.0%)	28 (21.9%)	100 (78.1%)	< 0.001^‡^
ARB	Yes	37 (3.5%)	6 (16.2%)	31 (83.8%)	< 0.001^‡^
Vitamin C	Yes	294 (27.7%)	174 (59.2%)	120 (40.8%)	0.126^‡^
Vitamin D	Yes	431 (40.5%)	236 (54.8%)	195 (45.2%)	0.724^‡^
Vitamin Zn	Yes	397 (37.3%)	264 (66.5%)	133 (33.5%)	< 0.001^‡^

*IVIg* Intravenous immunoglobulin, *NSAIDs* Non-steroidal anti-Inflammatory drugs, *ACEi* Angiotensin converting enzyme inhibitors, *ARB* Angiotensin II receptor blockers, *Zn* Zinc

^‡^: *P*-value conducted from Chi-square test

### Initial vital signs

Table 5 provides initial vital sign characteristics of COVID-19 patients, including heart rate, respiratory rate, temperature, blood pressure, oxygen therapy, and radiography test result. The findings shows that deceased patients had higher HR (83.03 bpm vs. 76.14 bpm, *P* < 0.001), lower RR (11.40 bpm vs. 16.25 bpm, *P* < 0.001), higher temperature (37.43 °C vs. 36.91 °C, *P* < 0.001), higher SBP (128.16 mmHg vs. 123.33 mmHg, *P* < 0.001), and higher O_2_ requirements (invasive: 75.0% vs. 25.0%, *P* < 0.001) compared to the survived patients. Additionally, deceased patients had higher MAP (99.35 mmHg vs. 96.08 mmHg, *P* = 0.005), and lower SPO_2_ percentage (81.29% vs. 91.95%, *P* < 0.001) compared to the survived patients. Furthermore, deceased patients had higher PEEP levels (5.83 cmH2O vs. 0.69 cmH2O, *P* < 0.001), higher FiO2 levels (51.43% vs. 8.97%, *P* < 0.001), and more frequent bilateral pneumonia (63.0% vs. 37.0%, *P* < 0.001) compared to the survived patients. There appears to be no relationship between diastolic blood pressure and treatment outcome (83.44 mmHg vs. 85.61 mmHg).

**Table 5:** Initial vital sign characteristics of patients infected with COVID-19

Initial vital sign data		Total (*n* = 1063)	Survived (*n* = 589)	Deceased (*n* = 474)	*P*-value
HR (Bpm)		79.21 ± 29.43	76.14 ± 18.65	83.03 ± 38.54	< 0.001^†^
RR (Bpm)		14.09 ± 4.84	16.25 ± 3.96	11.40 ± 4.48	< 0.001^†^
T (°C)		37.14 ± 1.06	36.91 ± 0.71	37.43 ± 1.32	< 0.001^†^
SBP (mmHg)		125.48 ± 20.88	123.33 ± 18.52	128.16 ± 23.23	< 0.001^†^
DBP (mmHg)		84.42 ± 17.86	83.45 ± 15.43	85.61 ± 20.43	0.050^†^
MAP (mmHg)		97.54 ± 18.97	96.08 ± 16.64	99.35 ± 21.40	0.005^†^
O_2_ therapy	Non-invasive	615 (57.9%)	477 (77.6%)	138 (22.4%)	< 0.001^‡^
	Invasive	448 (42.1%)	112 (25.0%)	336 (75.0%)	
O_2_ with mask (L/m)		4.12 ± 3.83	5.45 ± 3.19	2.47 ± 3.93	< 0.001^†^
Ventilator mode	SIMV	179 (16.8%)	24 (13.4%)	155 (86.6%)	< 0.001^‡^
	SPONT	76 (7.1%)	28 (36.8%)	48 (63.2%)	< 0.001^‡^
	CPAP	96 (9.0%)	36 (37.5%)	60 (62.5%)	
	BIPAP	93 (8.7%)	24 (25.8%)	69 (74.2%)	
	No	619 (58.2%)	477 (77.1%)	142 (22.9%)	
SPO_2_ (%)		87.19 ± 7.68	91.95 ± 4.09	81.29 ± 6.97	< 0.001^†^
PaO_2_ (%)		87.08 ± 5.92	90.81 ± 2.51	82.45 ± 5.67	< 0.001^†^
PEEP (cmH_2_O)		2.98 ± 4.01	0.69 ± 1.44	5.83 ± 4.33	< 0.001^†^
FiO_2_ (%)		27.91 ± 37.33	8.97 ± 18.91	51.43 ± 41.04	< 0.001^†^
Pneumonia	Unilateral	542 (51.0%)	396 (73.1%)	146 (26.9%)	< 0.001^†^
	Bilateral	521 (49.0%)	193 (37.0%)	328 (63.0%)	

*HR* Heart rate, *BPM* Beats per minute, *RR* Respiratory rate, *T* Temperatures, *SBP* Systolic blood pressure, *DBP* Diastolic blood pressure, *MAP* Mean arterial pressure, *SPO*_*2*_ Oxygen saturation, *PaO*_*2*_ Partial pressure of oxygen in the alveoli, *PEEP* Positive end-expiratory pressure, *FiO*_*2*_ Fraction of Inspired Oxygen, *Pneumonia* Radiography (X-ray) test result

^**†**^ *P*-value conducted from Independent t-test

^**‡**^ *P*-value conducted from Chi-square test

### Symptoms

Table 6 provides information on the symptoms of patients infected with COVID-19 by survival outcome. The table also shows the frequency of symptoms among patients. The most common symptom reported by patients was fever, which occurred in 67.0% of surviving and deceased patients. Dyspnea and nonproductive cough were the second and third most common symptoms, reported by 40.4% and 29.3% of the total sample, respectively. Other common symptoms listed in the Table were malodor (28.7%), dyspepsia (28.4%), and myalgia (25.6%).

**Table 6:** Symptoms of patients infected with COVID-19

Symptoms data		Total (*n* = 1063)	Survived (*n* = 589)	Deceased (*n* = 474)	*P*-value
Non-productive cough	Yes	311 (29.3%)	165 (53.1%)	146 (46.9%)	0.999^‡^
Productive cough	Yes	56 (5.3%)	12 (21.4%)	44 (78.6%)	< 0.001^‡^
Fever	Yes	240 (67.0%)	240 (67.0%)	118 (33.0%)	< 0.001^‡^
Chills	Yes	215 (20.2%)	121 (56.3%)	94 (43.7%)	0.774^‡^
Anorexia	Yes	105 (9.9%)	61 (58.1%)	44 (41.9%)	0.560^‡^
Myalgia	Yes	272 (25.6%)	119 (43.8%)	153 (56.3%)	< 0.001^‡^
Dyspnea	Yes	429 (40.4%)	164 (38.2%)	265 (61.8%)	< 0.001^‡^
Sore Throat	Yes	87 (8.2%)	30 (34.5%)	57 (65.5%)	< 0.001^‡^
Headache	Yes	167 (15.7%)	45 (26.9%)	122 (73.1%)	< 0.001^‡^
Dizziness	Yes	128 (12.0%)	58 (45.3%)	70 (54.7%)	0.014^‡^
Delirium	Yes	117 (11.0%)	42 (35.9%)	75 (64.1%)	< 0.001^‡^
Rhinorrhea	Yes	62 (5.8%)	24 (38.7%)	38 (61.3%)	0.006^‡^
Nasal congestion	Yes	69 (6.5%)	29 (42.0%)	40 (58.0%)	0.021^‡^
Olfactory	Yes	305 (28.7%)	219 (71.8%)	86 (28.2%)	< 0.001^‡^
Dyspepsia	Yes	302 (28.4%)	240 (79.5%)	62 (20.5%)	< 0.001^‡^
Nausea	Yes	208 (19.6%)	164 (78.8%)	44 (21.2%)	< 0.001^‡^
Vomiting	Yes	154 (14.5%)	136 (88.3%)	18 (11.7%)	< 0.001^‡^
Diarrhea	Yes	124 (11.7%)	72 (58.1%)	52 (41.9%)	0.527^‡^
Chest pain	Yes	62 (5.8%)	30 (48.4%)	32 (51.6%)	0.252^‡^
LOC	Yes	69 (6.5%)	20 (29.0%)	49 (71.0%)	< 0.001^‡^
Sepsis	Yes	88 (8.3%)	36 (40.9%)	52 (59.1%)	0.004^‡^
Respiratory failure	Yes	186 (17.5%)	57 (30.6%)	129 (69.4%)	< 0.001^‡^
Heart failure	Yes	106 (10.0%)	21 (19.8%)	85 (80.2%)	< 0.001^‡^
MODS	Yes	138 (13.0%)	12 (8.7%)	126 (91.3%)	< 0.001^‡^
Coagulopathy	Yes	52 (4.9%)	4 (7.7%)	48 (92.3%)	< 0.001^‡^
Secondary infection	Yes	112 (10.5%)	18 (16.1%)	94 (83.9%)	< 0.001^‡^
Stroke	Yes	32 (3.0%)	3 (9.4%)	29 (90.6%)	< 0.001^‡^
Hyperglycemia	Yes	15 (1.4%)	6 (40.0%)	9 (60.0%)	0.227^‡^
Acidosis	Yes	30 (2.8%)	5 (16.7%)	25 (83.3%)	< 0.001^‡^
I.C.U Admission	Yes	608 (57.2%)	257 (42.3%)	351 (57.7%)	< 0.001^‡^
I.C.U days		1.88 ± 2.61	0.98 ± 1.59	2.99 ± 3.16	< 0.001^†^

*Olfactory* Smell Disorders, *Dyspepsia* Indigestion, *LOC* Level of consciousness, *MODS* Multiple organ dysfunction syndrome, *Hemoptysis* Coughing up blood, *Coagulopathy* Bleeding disorder, *Hyperglycemia* High blood glucose, *ICU* Intensive care unit

^**†**^ *P*-value conducted from Independent t-test

^**‡**^ *P*-value conducted from Chi-square test

The *P*-values reported in the table show that some symptoms are significantly associated with death, including productive cough, dyspnea, sore throat, headache, delirium, olfactory symptoms, dyspepsia, nausea, vomiting, sepsis, respiratory failure, heart failure, MODS, coagulopathy, secondary infection, stroke, acidosis, and admission to the intensive care unit. Surviving and deceased patients also differed significantly in the average number of days spent in the ICU. There was no significant association between patient outcomes and symptoms such as nonproductive cough, chills, diarrhea, chest pain, and hyperglycemia.

### Laboratory tests

Table 7 shows the laboratory values of COVID-19 patients with the average values of the different laboratory results. The results show that the deceased patients had significantly lower levels of red blood cells (3.78 × 106/µL vs. 5.01 × 106/µL), hemoglobin (11.22 g/dL vs. 14.10 g/dL), and hematocrit (34.10% vs. 42.46%), whereas basophils and white blood cells did not differ significantly between the two groups. The percentage of neutrophils (65.59% vs. 62.58%) and monocytes (4.34% vs. 3.93%) was significantly higher in deceased patients, while the percentage of lymphocytes and eosinophils did not differ significantly between the two groups. In addition, deceased patients had higher levels of certain biomarkers, including D-dimer (1.347 mgFEU/L vs. 0.155 mgFEU/L), lactate dehydrogenase (174.61 U/L vs. 128.48 U/L), aspartate aminotransferase (93.09 U/L vs. 39.63 U/L), alanine aminotransferase (74.48 U/L vs. 28.70 U/L), alkaline phosphatase (119.51 IU/L vs. 81.34 IU/L), creatine phosphokinase-MB (4.65 IU/L vs. 3.33 IU/L), and positive troponin I (56.5% vs. 43.5%). The proportion of patients with positive C-reactive protein was also higher in the deceased group.

**Table 7:** Laboratory features of patients infected with COVID-19

Laboratory data		Total (*n* = 1063)	Survived (*n* = 589)	Deceased (*n* = 474)	*P*-value
RBC (× 10^6^/µL)		4.46 ± 1.70	5.01 ± 1.71	3.78 ± 1.43	< 0.001^†^
WBC (× 10^3^/µL)		8.71 ± 5.41	8.50 ± 4.78	8.97 ± 6.10	0.156^†^
Neutrophil (%)		64.25 ± 13.05	62.58 ± 15.83	65.59 ± 10.09	< 0.001^†^
Lymphocyte (%)		29.85 ± 14.64	29.64 ± 13.81	30.11 ± 15.62	0.597^†^
Monocyte (%)		4.11 ± 1.65	3.93 ± 1.66	4.34 ± 1.60	< 0.001^†^
Eosinophil (%)		2.39 ± 1.37	2.37 ± 1.43	2.41 ± 1.30	0.581^†^
Basophil (%)		0.49 ± 0.50	0.50 ± 0.50	0.48 ± 0.50	0.404^†^
Hb (g/dL)		12.82 ± 2.76	14.10 ± 2.02	11.22 ± 2.72	< 0.001^†^
HCT (%)		38.73 ± 7.87	42.46 ± 5.48	34.10 ± 7.93	< 0.001^†^
Alb (g/dL)		3.63 ± 1.14	3.55 ± 1.10	3.73 ± 1.17	0.013^†^
LDL (mg/dL)		119.89 ± 30.57	110.09 ± 26.24	132.06 ± 31.20	< 0.001^†^
HDL (mg/dL)		55.25 ± 21.50	58.52 ± 21.88	51.17 ± 20.31	< 0.001^†^
PT (seconds)		12.21 ± 2.22	12.59 ± 1.92	11.73 ± 2.45	< 0.001^†^
PTT (seconds)		31.71 ± 8.04	32.86 ± 7.12	30.28 ± 8.86	< 0.001^†^
INR (no unit)		0.98 ± 0.18	1.01 ± 0.15	0.94 ± 0.20	< 0.001^†^
ESR (mm/h)		13.27 ± 7.36	8.42 ± 5.26	19.30 ± 4.61	< 0.001^†^
CRP	+	335 (31.5%)	249 (74.3%)	86 (25.7%)	< 0.001^‡^
	+ +	293 (27.6%)	122 (41.6%)	171 (58.4%)	
	+ + +	204 (19.2%)	44 (21.6%)	160 (78.4%)	
	No	231 (21.7%)	174 (75.3%)	57 (24.7%)	
D-dimer (mg FEU/L)		0.687 ± 0.650	0.155 ± 0.052	1.347 ± 0.394	< 0.001^†^
LDH (U/L)		149.05 ± 40.65	128.48 ± 31.87	174.61 ± 35.58	< 0.001^†^
AST (U/L)		63.47 ± 47.93	39.63 ± 25.55	93.09 ± 52.53	< 0.001^†^
ALT (U/L)		49.11 ± 40.21	28.70 ± 17.53	74.48 ± 45.65	< 0.001^†^
ALK (IU/L)		98.36 ± 126.39	81.34 ± 92.85	119.51 ± 156.02	< 0.001^†^
CPK-MB (IU/L)		3.92 ± 3.07	3.33 ± 2.89	4.65 ± 3.14	< 0.001^†^
TNI	Positive	439 (41.3%)	191 (43.5%)	248 (56.5%)	< 0.001^‡^
	Negative	624 (58.7%)	398 (63.8%)	226 (36.2%)	
BUN (mg/dL)		23.49 ± 12.24	17.23 ± 6.39	31.27 ± 13.27	< 0.001^†^
Cr (mg/dL)		1.60 ± 1.31	0.98 ± 0.34	2.36 ± 1.62	< 0.001^†^
Na (mmol/L)		140.15 ± 7.22	139.18 ± 5.91	141.36 ± 8.42	< 0.001^†^
K (mmol/L)		4.57 ± 0.89	4.25 ± 0.67	4.95 ± 0.96	< 0.001^†^
Ca (mg/dL)		9.04 ± 0.76	9.02 ± 0.73	9.05 ± 0.79	0.508^†^
P (mg/dL)		3.45 ± 0.97	3.25 ± 0.91	3.70 ± 0.99	< 0.001^†^
Mg (mg/dL)		2.15 ± 0.57	2.16 ± 0.53	2.14 ± 0.60	0.643^†^
PLT (× 10^5^/µL)		2.55 ± 1.20	2.77 ± 1.13	2.27 ± 1.21	< 0.001^†^
TSH (mU/L)		2.17 ± 1.17	1.83 ± 0.81	2.59 ± 1.39	< 0.001^†^
T3 (ng/dL)		153.83 ± 26.55	157.43 ± 20.87	149.35 ± 31.71	< 0.001^†^
T4 (ng/dL)		7.93 ± 2.27	8.29 ± 1.93	7.48 ± 2.57	< 0.001^†^

*RBC* Red blood cell, *WBC* White blood cell, *LDL* Low-density lipoprotein, *Hb* Hemoglobin, *HCT* Hematocrit, *Alb* Albumin, *LDL* Low-density lipoprotein, *HDL* High-density lipoprotein, *PT* Prothrombin time, *PTT* Partial thromboplastin time, *INR* International normalized ratio, *ESR* Erythrocyte sedimentation rate, *CRP* C-reactive-protein, *LDH* Lactate dehydrogenase, *AST* Aspartate aminotransferase, *ALT* Alanine aminotransferase, *ALK* Alkaline phosphatase, *CPK-MB* Creatine phosphokinase-MB, *TNI* Troponin l, *BUN* Blood urea nitrogen, *Cr* Creatinine, *Na* Sodium, *K* Potassium, *Ca* Calcium, *P* Phosphorus, *Mg* Magnesium, *PLT* Platelet, *TSH* Thyroid stimulating hormone, *T3* Triiodothyronine, *T4* Thyroxine

^**†**^ *P*-value conducted from Independent t-test

^**‡**^ *P*-value conducted from Chi-square test

Other laboratory values with statistically significant differences between the two groups (*P* < 0.001) were INR, ESR, BUN, Cr, Na, K, P, PLT, TSH, T3, and T4. The surviving patients generally had lower values in these laboratory characteristics than the deceased patients.

### Model performance and evaluation

Five ML algorithms, namely DT, XGBoost, SVM, NB, and RF, were used in this study to build mortality prediction models COVID -19. The models were based on the optimal feature set selected in a previous step and were trained on the same data set. The effectiveness of the models was evaluated by calculating sensitivity, specificity, accuracy, F1 score, and AUC metrics. Table 8 shows the results of this performance evaluation. The average values are expressed from the test set as the mean (standard deviation).

**Table 8:** Performance comparison of ML models by feature sets in predicting mortality from COVID-19

Feature set	Model	Sensitivity	Specificity	Accuracy	F1-score	AUC
Demographic	DT	63.26 (13.59)	66.48 (12.19)	65.02 (2.60)	63.26 (13.59)	64.87 (2.24)
	XGBoost	60.50 (3.79)	68.30 (3.95)	64.83 (2.58)	60.50 (3.79)	64.40 (2.57)
	SVM	66.12 (3.15)	63.08 (3.59)	64.36 (1.67)	66.12 (3.15)	64.60 (1.48)
	NB	62.48 (3.77)	63.97 (5.08)	63.29 (2.33)	62.48 (3.77)	63.23 (2.10)
	RF	61.37 (2.57)	70.58 (3.96)	66.49 (1.84)	61.37 (2.57)	65.97 (1.77)
Clinical & Conditions	DT	90.85 (4.22)	86.04 (5.03)	88.24 (1.58)	90.85 (4.22)	88.45 (1.47)
	XGBoost	92.74 (2.46)	92.96 (2.37)	92.82 (0.77)	92.74 (2.46)	92.85 (0.70)
	SVM	90.70 (3.24)	92.61 (2.12)	91.72 (1.03)	90.70 (3.24)	91.66 (1.14)
	NB	88.40 (2.58)	94.32 (1.87)	91.63 (0.75)	88.40 (2.58)	91.36 (0.85)
	RF	92.88 (2.51)	92.85 (2.08)	92.82 (0.87)	92.88 (2.51)	92.86 (0.87)
Comorbidities	DT	74.83 (1.88)	79.19 (1.80)	77.35 (0.47)	74.83 (1.88)	77.01 (0.32)
	XGBoost	77.45 (1.32)	83.43 (1.28)	80.88 (0.57)	77.45 (1.32)	80.44 (0.74)
	SVM	74.83 (1.88)	78.93 (1.38)	77.19 (0.39)	74.83 (1.88)	76.88 (0.46)
	NB	75.58 (1.89)	79.76 (1.18)	77.98 (0.16)	78.58 (1.89)	77.67 (0.44)
	RF	75.19 (1.50)	81.96 (1.84)	79.08 (1.04)	75.19 (1.50)	78.58 (1.20)
Treatment	DT	75.17 (4.35)	87.62 (2.60)	81.94 (1.81)	75.17 (4.35)	81.39 (1.90)
	XGBoost	78.42 (3.43)	89.27 (1.58)	84.29 (1.59)	78.42 (3.43)	83.84 (1.69)
	SVM	72.89 (2.39)	91.15 (1.34)	82.82 (1.30)	72.89 (2.39)	82.02 (1.36)
	NB	72.33 (3.64)	88.50 (2.25)	81.13 (2.04)	72.33 (3.64)	80.42 (2.10)
	RF	79.17 (3.32)	89.55 (1.36)	84.80 (1.52)	79.17 (3.32)	84.36 (1.61)
Initial vital signs	DT	90.30 (2.52)	98.53 (1.91)	95.77 (1.11)	92.30 (2.52)	95.42 (1.17)
	XGBoost	95.85 (1.97)	99.83 (0.53)	98.06 (0.97)	95.85 (1.97)	97.84 (1.06)
	SVM	94.45 (1.61)	99.49 (0.62)	97.24 (1.02)	94.45 (1.61)	96.97 (1.06)
	NB	87.37 (2.09)	99.21 (0.76)	93.95 (1.19)	87.37 (2.09)	93.29 (1.24)
	RF	94.63 (2.02)	99.83 (0.54)	97.52 (1.12)	94.63 (2.02)	97.23 (1.20)
Symptoms	DT	92.79 (3.69)	97.09 (1.39)	95.24 (2.05)	92.79 (3.69)	94.94 (2.25)
	XGBoost	97.08 (1.32)	98.76 (0.79)	98.03 (0.78)	97.08 (1.32)	97.92 (0.78)
	SVM	91.78 (2.48)	98.02 (1.05)	95.27 (1.38)	91.78 (2.48)	94.90 (1.45)
	NB	82.03 (4.83)	90.19 (3.29)	86.58 (2.19)	82.03 (4.83)	86.11 (2.32)
	RF	95.55 (2.17)	97.82 (0.51)	96.83 (1.01)	95.55 (2.17)	96.69 (1.14)
Laboratory test	DT	100 (0.0)	100 (0.0)	100 (0.0)	100 (0.0)	100 (0.0)
	XGBoost	100 (0.0)	100 (0.0)	100 (0.0)	100 (0.0)	100 (0.0)
	SVM	99.80 (0.33)	100 (0.0)	99.91 (0.15)	99.80 (0.33)	99.90 (0.16)
	NB	100 (0.0)	100 (0.0)	100 (0.0)	100 (0.0)	100 (0.0)
	RF	100 (0.0)	100 (0.0)	100 (0.0)	100 (0.0)	100 (0.0)

The average values are expressed from the test set as the Mean (SD)

*DT* Decision Tree, *XGBoost* eXtreme Gradient Boosting, *SVM* Support Vector Machine, *NB* Naïve Bayes, *RF* Random Forest

The results show that the performance of the models varies widely in the different feature categories. The Laboratory Tests category achieved the highest performance, with all models scoring 100% in all metrics. The Symptoms and initial Vital Signs categories also show high performance, with XGBoost achieving the highest accuracy of 98.03% and DT achieving the highest sensitivity of 92.79%.

The Clinical and Conditions category also showed high performance, with all models showing accuracy above 91%. XGBoost achieved the highest sensitivity and specificity of 92.74% and 92.96%, respectively. In contrast, the Demographics category showed the lowest performance, with all models achieving less than 66.5% accuracy.

In summary, the results suggest that certain feature categories may be more useful than others in predicting mortality from COVID-19 and that some ML models may perform better than others depending on the feature category used.

### Feature importance

SHapley Additive exPlanations (SHAP) values indicate the importance or contribution of each feature in predicting model output. These values help to understand the influence and importance of each feature on the model's decision-making process.

**Fig. 2:**
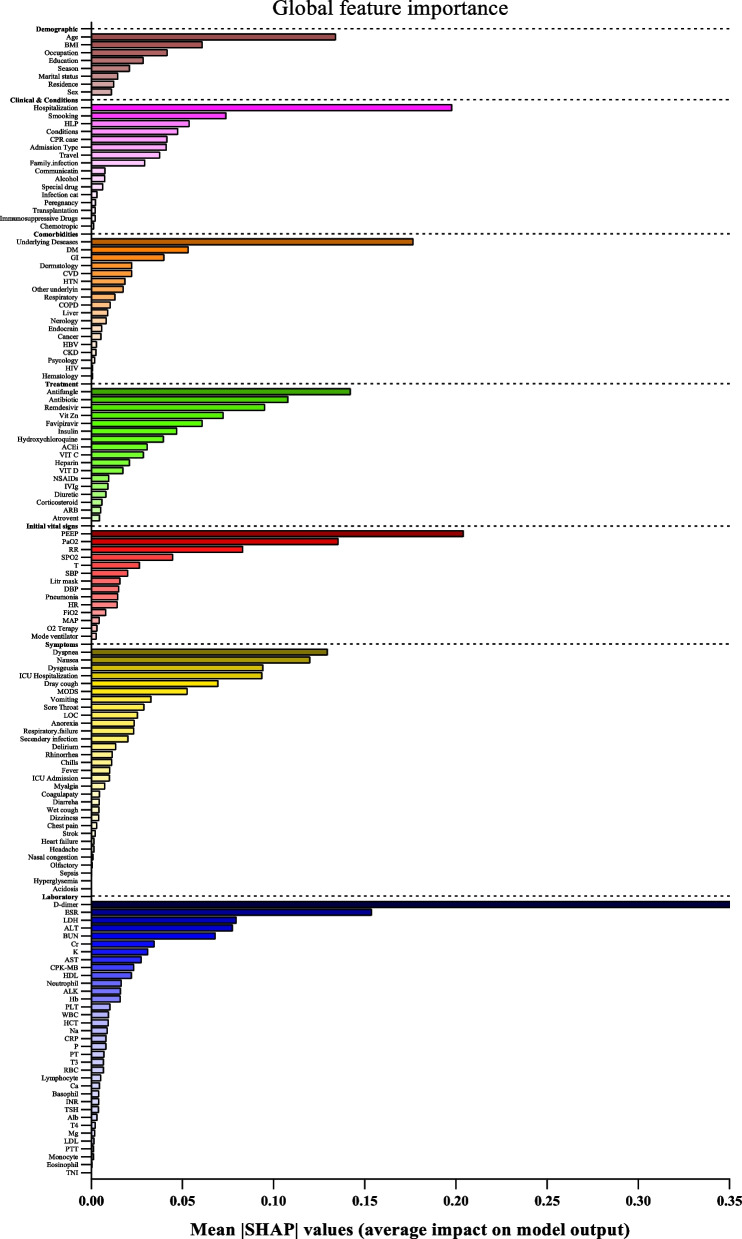
Feature importance based on SHAP-values. The mean absolute SHAP values are depicted, to illustrate global feature importance. The SHAP values change in the spectrum from dark (higher) to light (lower) color

In Fig. 2, the mean absolute SHAP values are shown to depict global feature importance. Figure 2 shows the contribution of each feature within its respective group as calculated by the XGBoost prediction model using SHAP. According to the SHAP method, the features that had the greatest impact on predicting COVID-19 mortality were, in descending order: D-dimer, CPR, PEEP, underlying disease, ESR, antifungal treatment, PaO2, age, dyspnea, and nausea.

**Fig. 3:**
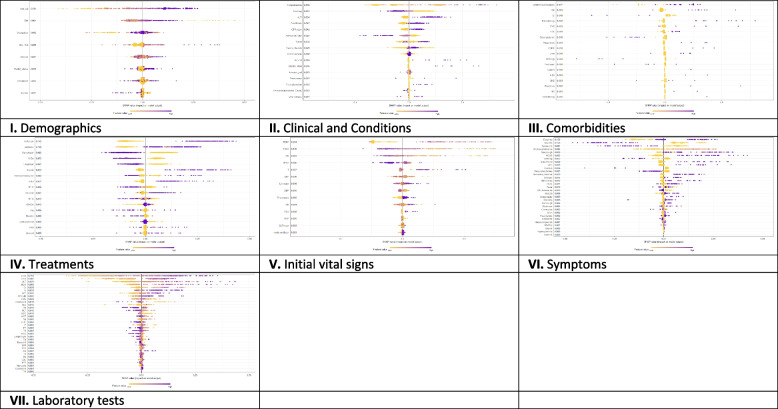
The SHAP-based feature importance of all categories (I to VII) for COVID‑19 mortality prediction, calculated with the XGBoost model. The local explanatory summary shows the direction of the relationship between a feature and patient outcome. Positive SHAP values indicate death, whereas negative SHAP values indicate survival. As the color scale shows, higher values are blue while lower values are orenge

On the other hand, Fig. 3 presents the local explanation summary that indicates the direction of the relationship between a variable and COVID-19 outcome. As shown in Fig. 3(I to VII), older age and very low BMI were the two demographic factors with the greatest impact on model outcome, followed by clinical factors such as higher CPR, hospitalization, and hyperlipidemia. Higher mortality rates were associated with patients who smoked and had traveled in the past 14 days. Patients with underlying diseases, especially HTN, died more frequently. In contrast, the use of remdesivir, Vit Zn, and favipiravir is associated with lower mortality. Initial vital signs such as high PEEP, low PaO2 and RR had the greatest impact, as did symptoms such as dyspnea, MODS, sore throat and LOC. A higher risk of mortality is observed in patients with higher D-dimer levels and ESR as the most consequential laboratory tests, followed by K, AST and CPK-MB.

**Fig. 4:**
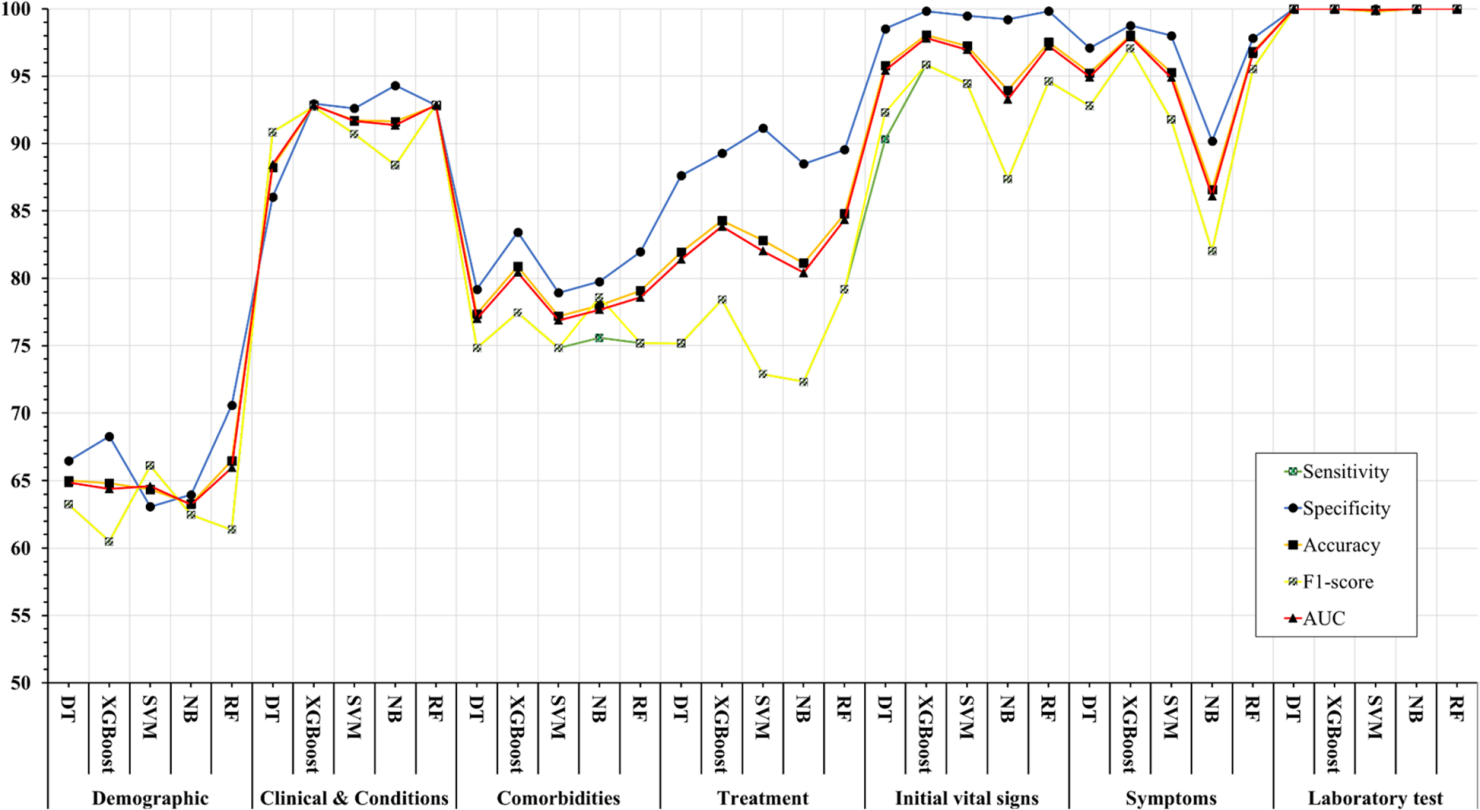
Association between feature sets and performance of machine learning algorithms in predicting COVID-19’s mortality

Using the feature types listed in Appendix Table 1, Fig. 4 shows that the performance of ML algorithms can be improved by increasing the number of features used in training, especially in distinguishing between symptoms, comorbidities, and treatments. In addition, the amount and quality of data used for training can significantly affect algorithm performance, with laboratory tests being more informative than initial vital signs. Regarding the influence of features, quantitative features tend to have a more positive effect on performance than qualitative features; clinical conditions tend to be more informative than demographic data. Thus, both the amount of data and the type of features used have a significant impact on the performance of ML algorithms.

## Discussion

The COVID-19 pandemic has presented unprecedented public health challenges worldwide and requires a deep understanding of the factors contributing to COVID-19 mortality to enable effective management and intervention. This study used machine learning analysis to uncover the predictive power of an extensive dataset that includes wide range of personal, clinical, preclinical, and laboratory variables associated with COVID-19 mortality.

This study confirms previous research on COVID-19 outcomes that highlighted age as a significant predictor of mortality [45–47], along with comorbidities such as hypertension and diabetes [48, 49]. Underlying conditions such as cardiovascular and renal disease also contribute to mortality risk [50, 51].

Regarding treatment, antibiotics, remdesivir, favipiravir, and vitamin zinc are associated with lower mortality [52, 53], whereas heparin, insulin, antifungals, ACE, and ARBs are associated with higher mortality [54]. This underscores the importance of drug choice in COVID -19 treatment.

Initial vital signs such as heart rate, respiratory rate, temperature, and oxygen therapy differ between surviving and deceased patients [55]. Deceased patients often have increased heart rate, lower respiratory rate, higher temperature, and increased oxygen requirements, which can serve as early indicators of disease severity.

Symptoms such as productive cough, dyspnea, and delirium are significantly associated with COVID-19 mortality, emphasizing the need for immediate monitoring and intervention [56]. Laboratory tests show altered hematologic and biochemical markers in deceased patients, underscoring the importance of routine laboratory monitoring in COVID-19 patients [57, 58].

The ML algorithms were used in the study to predict mortality COVID-19 based on these multilayered variables. XGBoost and Random Forest performed better than other algorithms and had high recall, specificity, accuracy, F1 score, and AUC. This highlights the potential of ML, particularly the XGBoost algorithm, in improving prediction accuracy for COVID-19 mortality [59]. The study also highlighted the importance of drug choice in treatment and the potential of ML algorithms, particularly XGBoost, in improving prediction accuracy. However, the study's findings differ from those of Moulaei [60], Nopour [61], and Mehraeen [62] in terms of the best-performing ML algorithm and the most influential variables. While Moulaei [60] found that the random forest algorithm had the best performance, Nopour [61] and Ikemura [63] identified the artificial neural network and stacked ensemble models, respectively, as the most effective. Additionally, the most influential variables in predicting mortality varied across the studies, with Moulaei [60] highlighting dyspnea, ICU admission, and oxygen therapy, and Ikemura [63] identifying systolic and diastolic blood pressure, age, and other biomarkers. These differences may be attributed to variations in the datasets, feature selection, and model training.

However, it is important to note that the choice of algorithm should be tailored to the specific dataset and research question. In addition, the results suggest that a comprehensive approach that incorporates different feature categories may lead to more accurate prediction of COVID-19 mortality. In general, the results suggest that the performance of ML models is influenced by the number and type of features in each category. While some models consistently perform well across different categories (e.g., XGBoost), others perform better for specific types of features (e.g., SVM for Demographics).

Analysis of the importance of characteristics using SHAP values revealed critical factors affecting model results. D-dimer values, CPR, PEEP, underlying diseases, and ESR emerged as the most important features, highlighting the importance of these variables in predicting COVID-19 mortality. These results provide valuable insights into the underlying mechanisms and risk factors associated with severe COVID-19 outcomes.

The types of features used in ML models fall into two broad categories: quantitative (numerical) and qualitative (binary or categorical). The performance of ML methods can vary depending on the type of features used. Some algorithms work better with quantitative features, while others work better with qualitative features. For example, decision trees and random forests work well with both types of features [64], while neural networks often work better with quantitative features [65, 66]. Accordingly, we consider these levels for the features under study to better assess the impact of the data.

The success of ML algorithms depends largely on the quality and quantity of the data on which they are trained [67–69]. Recent research, including the 2021 study by Sarker IH. [26], has shown that a larger amount of data can significantly improve the performance of deep learning algorithms compared to traditional machine learning techniques. However, it should be noted that the effect of data size on model performance depends on several factors, such as data characteristics and experimental design. This underscores the importance of carefully and judiciously selecting data for training.

### Limitations

One of the limitations of this study is that it relies on data collected from a single hospital in Abadan, Iran. The data may not be representative of the diversity of COVID -19 cases in different regions, and there may be differences in data quality and completeness. In addition, retrospectively collected data may have biases and inaccuracies. Although the study included a substantial number of COVID -19 patients, the sample size may still limit the generalizability of the results, especially for less common subgroups or certain demographic characteristics.

### Future works

Future studies could adopt a multi-center approach to improve the scope and depth of research on COVID-19 outcomes. This could include working with multiple hospitals in different regions of Iran to ensure a more diverse and representative sample. By conducting prospective studies, researchers can collect data in real time, which reduces the biases associated with retrospective data collection and increases the reliability of the results. Increasing sample size, conducting longitudinal studies to track patient progression, and implementing quality assurance measures are critical to improving generalizability, understanding long-term effects, and ensuring data accuracy in future research efforts. Collectively, these strategies aim to address the limitations of individual studies and make an important contribution to a more comprehensive understanding of COVID-19 outcomes in different populations and settings.

## Conclusions

In summary, this study demonstrates the potential of ML algorithms in predicting COVID-19 mortality based on a comprehensive set of features. In addition, the interpretability of the models using SHAP-based feature importance, which revealed the variables strongly correlated with mortality. This study highlights the power of data-driven approaches in addressing critical public health challenges such as the COVID-19 pandemic. The results suggest that the performance of ML models is influenced by the number and type of features in each feature set. These findings may be a valuable resource for health professionals to identify high-risk patients COVID-19 and allocate resources effectively.

### Supplementary Information

**Supplementary Material 1.**

## Data Availability

The datasets used and/or analyzed during the current study are available from the corresponding author on reasonable request.

## Abbreviations

WHO: World Health Organization
MERS: Middle east respiratory syndrome
SARS: Severe acute respiratory syndrome
RT-PCR: Reverse transcription polymerase chain reaction
PSM: Propensity score matching
SMOTE: Synthetic minority over-sampling technique
MCAR: Missing completely at random
DT: Decision tree
XGBoost: EXtreme gradient boosting
SVM: Support vector machine
NB: Naïve bayes
RF: Random forest
CV: Cross-validation
TP: True positive
TN: True negative
FP: False positive
FN: False negative
ML: Machine learning
AI: Artificial Intelligence
SHAP: Shapely additive explanation
CPR: Cardiopulmonary Resuscitation
HTN: Hypertension
DM: Diabetes mellitus
CVD: Cardiovascular disease
CKD: Chronic Kidney disease
COPD: Chronic obstructive pulmonary disease
HIV: Human immunodeficiency virus
HBV: Hepatitis B virus
Respiratory: Such as influenza, pneumonia, asthma, bronchitis, and chronic obstructive airways disease
GI: Gastrointestinal
Neurology: Such as epilepsy, learning disabilities, neuromuscular disorders, autism, ADD, brain tumors, and cerebral palsy
Liver: Such as fatty liver disease and cirrhosis
Hematology: Blood disease
Dermatology: Skin diseases
Psychology: Mental disorders
IVIg: Intravenous immunoglobulin
NSAIDs: Non-steroidal anti-Inflammatory drugs
ACEi: Angiotensin converting enzyme inhibitors
ARB: Angiotensin II receptor blockers
Zn: Zinc
HR: Heart rate
BPM: Beats per minute
RR: Respiratory rate
T: Temperatures
SBP: Systolic blood pressure
DBP: Diastolic blood pressure
MAP: Mean arterial pressure
SPO2: Oxygen saturation
PaO2: Partial pressure of oxygen in the alveoli
PEEP: Positive end-expiratory pressure
FiO2: Fraction of inspired oxygen
Pneumonia: Radiography (X-ray) test result
Olfactory: Smell disorders
Dyspepsia: Indigestion
LOC: Level of consciousness
MODS: Multiple organ dysfunction syndrome
Hemoptysis: Coughing up blood; Coagulopathy: bleeding disorder
Hyperglycemia: High blood glucose
ICU: Intensive care unit
RBC: Red blood cell
WBC: White blood cell
LDL: Low-density lipoprotein
Hb: Hemoglobin
HCT: Hematocrit
Alb: Albumin
LDL: Low-density lipoprotein
HDL: High-density lipoprotein
PT: Prothrombin time
PTT: Partial thromboplastin time
INR: International normalized ratio
ESR: Erythrocyte sedimentation rate
CRP: C-reactive-protein
LDH: Lactate dehydrogenase
AST: Aspartate aminotransferase
ALT: Alanine aminotransferase
ALK: Alkaline phosphatase
CPK-MB: Creatine phosphokinase-MB
TNI: Troponin I
BUN: Blood urea nitrogen
Cr: Creatinine
Na: Sodium
K: Potassium
Ca: Calcium
P: Phosphorus
Mg: Magnesium
PLT: Platelet
TSH: Thyroid stimulating hormone
T3: Triiodothyronine
T4: Thyroxine
