# Supplementary material for: The predictive power of data: machine learning analysis for Covid-19 mortality based on personal, clinical, preclinical, and laboratory variables in a case–control study
Source: BMC Infect Dis. 2024 Apr 18;24:411. doi: 10.1186/s12879-024-09298-w (PMC11025285; doi:10.1186/s12879-024-09298-w)
Supplement: Supplementary file 1 — Supplementary Material 1. [file 12879_2024_9298_MOESM1_ESM.docx]

| **Appendix table 1.** Primary features recorded in the COVID‑19 hospital registry database, organized by eight class data. | | | | | | | | | |
| --- | --- | --- | --- | --- | --- | --- | --- | --- | --- |
| **No.** | **Features name** | **Features type** | | | **No.** | **Features name** | **Features type** | | |
|  |  | **Continuous** | **Categorical** | **Binary** |  |  | **Continuous** | **Categorical** | **Binary** |
| **I. Demographic data** | | | | | **VI. Symptoms data** | | | | |
| 1 | Age |  |  |  | 74 | Non-productive cough |  |  |  |
| 2 | Sex |  |  |  | 75 | Productive cough |  |  |  |
| 3 | Occupation |  |  |  | 76 | Fever |  |  |  |
| 4 | Place of residence |  |  |  | 77 | Chills |  |  |  |
| 5 | Marital status |  |  |  | 78 | Anorexia |  |  |  |
| 6 | Education level |  |  |  | 79 | Myalgia |  |  |  |
| 7 | BMI (kg/cm^2^) |  |  |  | 80 | Dyspnea |  |  |  |
| 8 | Season of admission |  |  |  | 81 | Sore Throat |  |  |  |
| **II. Clinical & Conditions data** | | | | | 82 | Headache |  |  |  |
| 9 | Hospitalization, day |  |  |  | 83 | Dizziness |  |  |  |
| 10 | Admission type |  |  |  | 84 | Delirium |  |  |  |
| 11 | No. of infection |  |  |  | 85 | Rhinorrhea |  |  |  |
| 12 | Family infection |  |  |  | 86 | Nasal congestion |  |  |  |
| 13 | Travel |  |  |  | 87 | Olfactory |  |  |  |
| 14 | Communication |  |  |  | 88 | Dyspepsia |  |  |  |
| 15 | CPR case |  |  |  | 89 | Nausea |  |  |  |
| 16 | Underlying conditions |  |  |  | 90 | Vomiting |  |  |  |
| 17 | Hyperlipidemia |  |  |  | 91 | Diarrhea |  |  |  |
| 18 | Alcohol consumption |  |  |  | 92 | Chest pain |  |  |  |
| 19 | Transplantation |  |  |  | 93 | LOC |  |  |  |
| 20 | Chemotropic |  |  |  | 94 | Sepsis |  |  |  |
| 21 | Special Drugs |  |  |  | 95 | Respiratory failure |  |  |  |
| 22 | Immunosuppressive Drugs |  |  |  | 96 | Heart failure |  |  |  |
| 23 | Pregnancy |  |  |  | 97 | MODS |  |  |  |
| 24 | Smoking |  |  |  | 98 | Coagulopathy |  |  |  |
| **III. Comorbidities data** | | | | | 99 | Secondary infection |  |  |  |
| 25 | Comorbidity |  |  |  | 100 | Stroke |  |  |  |
| 26 | HTN |  |  |  | 101 | Hyperglycemia |  |  |  |
| 27 | DM |  |  |  | 102 | Acidosis |  |  |  |
| 28 | CVD |  |  |  | 103 | I.C.U Admission |  |  |  |
| 29 | CKD |  |  |  | 104 | I.C.U days |  |  |  |
| 30 | COPD |  |  |  | **VII. Laboratory tests data** | | | | |
| 31 | HIV |  |  |  | 105 | RBC (×10^6^/µL) |  |  |  |
| 32 | HBV |  |  |  | 106 | WBC (×10^3^/µL) |  |  |  |
| 33 | Cancer |  |  |  | 107 | Neutrophil (%) |  |  |  |
| 34 | Respiratory |  |  |  | 108 | Lymphocyte (%) |  |  |  |
| 35 | GI |  |  |  | 109 | Monocyte (%) |  |  |  |
| 36 | Neurology |  |  |  | 110 | Eosinophil (%) |  |  |  |
| 37 | Endocrine |  |  |  | 111 | Basophil (%) |  |  |  |
| 38 | Liver |  |  |  | 112 | Hb (g/dL) |  |  |  |
| 39 | Hematology |  |  |  | 113 | HCT (%) |  |  |  |
| 40 | Dermatology |  |  |  | 114 | Alb (g/dL) |  |  |  |
| 41 | Psychology |  |  |  | 115 | LDL (mg/dL) |  |  |  |
| 42 | Other diseases |  |  |  | 116 | HDL (mg/dL) |  |  |  |
| **IV. Treatment data** | | | | | 117 | PT (seconds) |  |  |  |
| 43 | Antibiotic |  |  |  | 118 | PTT (seconds) |  |  |  |
| 44 | Remdesivir |  |  |  | 119 | INR (no unit) |  |  |  |
| 45 | Favipiravir |  |  |  | 120 | ESR (mm/h) |  |  |  |
| 46 | Hydroxychloroquine |  |  |  | 121 | CRP |  |  |  |
| 47 | Heparin |  |  |  | 122 | D-dimer (mg FEU/L) |  |  |  |
| 48 | Atrovent |  |  |  | 123 | LDH (U/L) |  |  |  |
| 49 | Insulin |  |  |  | 124 | AST (U/L) |  |  |  |
| 50 | Diuretic |  |  |  | 125 | ALT (U/L) |  |  |  |
| 51 | Antifungal |  |  |  | 126 | ALK (IU/L) |  |  |  |
| 52 | Corticosteroid |  |  |  | 127 | CPK-MB (IU/L) |  |  |  |
| 53 | IVIg |  |  |  | 128 | TNI |  |  |  |
| 54 | NSAIDs |  |  |  | 129 | BUN (mg/dL) |  |  |  |
| 55 | ACEi |  |  |  | 130 | Cr (mg/dL) |  |  |  |
| 56 | ARB |  |  |  | 131 | Na (mmol/L) |  |  |  |
| 57 | Vitamin C |  |  |  | 132 | K (mmol/L) |  |  |  |
| 58 | Vitamin D |  |  |  | 133 | Ca (mg/dL) |  |  |  |
| 59 | Vitamin Zn |  |  |  | 134 | P (mg/dL) |  |  |  |
| **V. Initial vital signs data** | | | | | 135 | Mg (mg/dL) |  |  |  |
| 60 | HR (Bpm) |  |  |  | 136 | PLT (×10^5^/µL) |  |  |  |
| 61 | RR (Bpm) |  |  |  | 137 | TSH (mU/L) |  |  |  |
| 62 | T (°C) |  |  |  | 138 | T3 (ng/dL) |  |  |  |
| 63 | SBP (mmHg) |  |  |  | 139 | T4 (ng/dL) |  |  |  |
| 64 | DBP (mmHg) |  |  |  | **VIII. Output data** | | | | |
| 65 | MAP (mmHg) |  |  |  | 140 | Patients group |  |  |  |
| 66 | O_2_ therapy |  |  |  |  |  |  |  |  |
| 67 | O_2_ with mask (L/m) |  |  |  |  |  |  |  |  |
| 68 | Ventilator mode |  |  |  |  |  |  |  |  |
| 69 | SPO_2_ (%) |  |  |  |  |  |  |  |  |
| 70 | PaO_2_ (%) |  |  |  |  |  |  |  |  |
| 71 | PEEP (cmH_2_O) |  |  |  |  |  |  |  |  |
| 72 | FiO_2_ (%) |  |  |  |  |  |  |  |  |
| 73 | Pneumonia |  |  |  |  |  |  |  |  |
